# Trajectories of Emotion Recognition Training in Virtual Reality and Predictors of Improvement for People with a Psychotic Disorder

**DOI:** 10.1089/cyber.2022.0228

**Published:** 2023-04-14

**Authors:** Saskia A. Nijman, Wim Veling, Marieke E. Timmerman, Gerdina H.M. Pijnenborg

**Affiliations:** ^1^ Department of Long-Term Care, GGZ Drenthe, Assen, Netherlands.; ^2^ University Medical Center Groningen, University of Groningen, Groningen, Netherlands.; ^3^ Department of Clinical & Developmental Neuropsychology and Faculty of Behavioral and Social Sciences, University of Groningen, Groningen, Netherlands.; ^4^ Department of Psychometrics and Statistics, Faculty of Behavioral and Social Sciences, University of Groningen, Groningen, Netherlands.

**Keywords:** social cognition training, cognitive remediation, social cognition, schizophrenia, facial affect recognition, emotion perception

## Abstract

Meta-analyses have found that social cognition training (SCT) has large effects on the emotion recognition ability of people with a psychotic disorder. Virtual reality (VR) could be a promising tool for delivering SCT. Presently, it is unknown how improvements in emotion recognition develop during (VR-)SCT, which factors impact improvement, and how improvements in VR relate to improvement outside VR. Data were extracted from task logs from a pilot study and randomized controlled trials on VR-SCT (*n* = 55). Using mixed-effects generalized linear models, we examined the: (a) effect of treatment session (1–5) on VR accuracy and VR response time for correct answers; (b) main effects and moderation of participant and treatment characteristics on VR accuracy; and (c) the association between baseline performance on the Ekman 60 Faces task and accuracy in VR, and the interaction of Ekman 60 Faces change scores (i.e., post-treatment − baseline) with treatment session. Accounting for the task difficulty level and the type of presented emotion, participants became more accurate at the VR task (*b* = 0.20, *p* < 0.001) and faster (*b* = −0.10, *p* < 0.001) at providing correct answers as treatment sessions progressed. Overall emotion recognition accuracy in VR decreased with age (*b* = −0.34, *p* = 0.009); however, no significant interactions between any of the moderator variables and treatment session were found. An association between baseline Ekman 60 Faces and VR accuracy was found (*b* = 0.04, *p* = 0.006), but no significant interaction between difference scores and treatment session. Emotion recognition accuracy improved during VR-SCT, but improvements in VR may not generalize to non-VR tasks and daily life.

## Introduction

Deficits in social cognition, among which problems in emotion perception, are common in people with a psychotic disorder^1^ and predict problems in social functioning, for example,^2,3^ Social Cognition Training (SCT) combines compensatory strategy training with repeated practice with social stimuli. Meta-analyses^4–7^ have found large effects of SCT on emotion perception. In the past years, studies have explored using Virtual Reality (VR) to train social cognition.^8–10^

Compared with conventional SCT, VR-SCT has the potential added benefit of facilitating practice in an interactive, dynamic and realistic environment. Two^8,10^ preliminary studies observed moderate to large improvement in emotion perception. However, a randomized controlled trial (RCT; *n* = 83) by our research group, comparing a VR-SCT, “DiSCoVR” (Dynamic Interactive Social Cognition Training in Virtual Reality), with an active control condition (VR relaxation) showed no effects on emotion perception or Theory of Mind.^11^

Given that this intervention was based on effective protocols and findings from previous meta-analyses of SCT, it was unclear why no effect was found; this could be due to ineffectiveness of the protocol in general, or due to a lack of generalization of VR improvements to non-VR measures.

In fact, little is known *in general* about how SCT works, and for whom; to our knowledge, only meta-analyses have investigated this issue, with inconsistent results.^6,8,12^ Moreover, SCT studies to date only report pre- and post-treatment emotion perception scores. These demonstrate improvements *after* SCT, but neither reveal which factors are relevant for improvement, nor what happens *during* SCT. Cella and Wykes^13^ investigated treatment processes during Cognitive Remediation Training, a form of cognitive training similar to SCT, but aimed at neurocognition (e.g., memory).

The therapeutic alliance during training predicted improvements in functioning, whereas other variables (e.g., number of tasks completed) predicted gains in neurocognition. The only study to report data during SCT is a single case study of a person with traumatic brain injury,^14^ showing session-by-session improvement of emotion perception accuracy, particularly for negative emotions. After starting treatment, an increase in response time occurred, trending downward as sessions progressed.

Thus, how improvements following SCT develop over time, and which factors are relevant for treatment success, is still largely unknown. However, it is particularly relevant to understand why SCT might (or might not) be effective, as session-by-session measurements can demonstrate when and how improvements occur.

Computerized interventions could provide a unique opportunity to track this treatment process, as several parameters can be logged that might be more difficult to track in analog exercises (e.g., reaction time). VR also provides an immersive, interactive setting with control of its parameters (e.g., difficulty) and its content,^15,16^ in which behavior can be recorded unobtrusively. For example, Freeman et al.^17^ used VR to treat persecutory delusions and tracked movement in VR.

Participants receiving cognitive behavioral therapy moved a greater distance (i.e., explored the environment more) than people receiving only exposure. However, tracking parameters to examine treatment processes during VR exercises has not been used for SCT. Moreover, the relationship between data recorded during VR treatment and improvement outside VR has, to our knowledge, not been investigated.

In this study, we investigate the treatment process and potentially relevant factors for treatment success. Given the lack of effects of DiSCoVR on social cognition measures, we investigated whether a treatment effect was present in VR, and whether this potential effect generalized. Given the dearth of knowledge on factors contributing to treatment efficacy, we opted to use an exploratory approach, selecting variables from our data that have previously been investigated as predictors of emotion recognition^18–20^ and moderators of SCT efficacy.^6,8,12^ We investigated the following research questions:

(1) (a) Does emotion perception accuracy during a VR SCT training improve over time, while accounting for the difficulty level and the type of emotion shown?
  (b)  Do participants become faster at correctly identifying emotions during a VR training, while accounting for the difficulty level and the type of emotion shown?
(2) (a) Do participant characteristics (i.e., age, gender, baseline neurocognition, education level, premorbid intelligence, number of hospitalizations, baseline emotion perception, baseline symptoms) predict *changes* in emotion perception accuracy over time during VR-SCT sessions?
  (b)  Do treatment characteristics (i.e., strategy use, duration of VR practice, duration of sessions, time taken to complete treatment) predict *changes* in emotion perception accuracy over time during VR-SCT sessions?
(3) Is (improvement in) accuracy in the VR environment associated with accuracy on a conventional task of emotion perception?

## Materials and Methods

### Design

This study combines data from two studies: a single-group, uncontrolled pilot study and a single-blind RCT on DiSCoVR, a VR-SCT, as the emotion recognition module was nearly identical in both studies. For detailed information on these studies and DiSCoVR, cf. Refs.^8,11,21^

### Participants

#### Inclusion criteria

- Age 18–65.
- Diagnosis of psychotic disorder, determined in the past 3 years with a structured diagnostic instrument or verified at baseline using the Mini-International Psychiatric Interview.^22^
- Social cognitive deficits as indicated by a referring clinician.

#### Exclusion criteria

- A relevant neurological disorder, such as dementia (pilot study only).
- Substance dependence (pilot study only).
- (Photosensitive) Epilepsy.
- Inadequate proficiency of Dutch language.
- A diagnosis of an intellectual disability and/or estimated IQ under 70.

Participants were recruited through clinical referral and self-enrollment from mental health institutions in Netherlands: University Medical Center Groningen (both studies), GGZ Drenthe (both studies), GGZ Delfland (both studies), Zeeuwse Gronden (RCT), and GGZ Westelijk Noord-Brabant (RCT).

### Intervention

DiSCoVR consisted of 16 individual 45–60 minutes, twice weekly one-on-one treatment sessions, guided by a trained therapist. The intervention was modeled after existing, effective SCT protocols (e.g., Roberts^23^ and Westerhof-Evers et al.^24^) and was designed to gradually increase the complexity of training content. Sessions consisted of face-to-face discussion (e.g., about goals and strategies) and practice with social stimuli in VR.

The intervention encompassed three modules, each targeting a different domain of social cognition (i.e., emotion recognition; theory of mind and social perception; and social interaction). The latter two modules will be described only briefly, as only data from the first module was used. The VR environments were developed by CleVR BV.

**FIG. 1.**
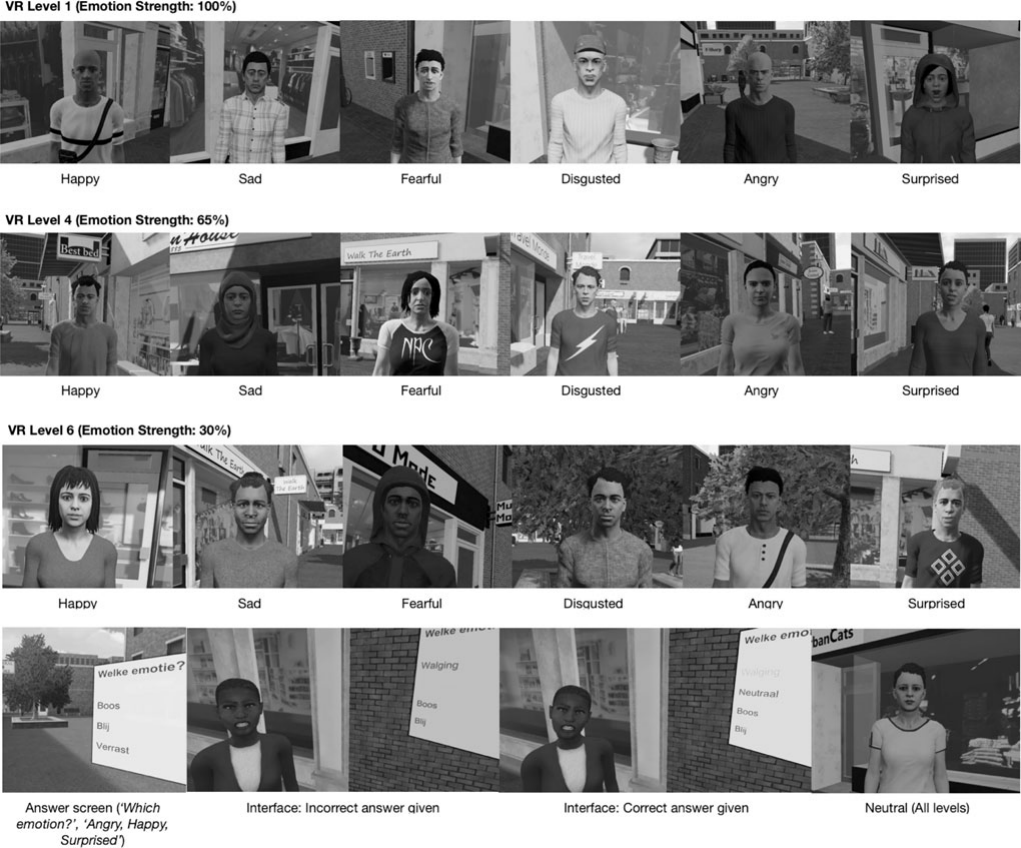
Screenshots of VR environment (emotions and interface). VR, virtual reality.

Module 1 (sessions 1–5) targeted emotion recognition. Personal social goals (e.g., “Having a conversation with a stranger”) were established, and compensatory strategies for emotion recognition were introduced (Supplementary Appendix SA1). A strategy was selected before every VR practice session. Participants were encouraged to use these in daily life (Supplementary Appendix SA2). In VR, participants encountered stationary characters (“avatars”) in a shopping street (Fig. 1).

On approach, avatars showed one of six basic emotions (happiness, sadness, fear, disgust, anger or surprise) or no emotion (neutral). Participants selected the emotion shown from a multiple-choice menu. For incorrect answers, the avatar expressed the emotion again more strongly and participants could try again. These emotions were based on the facial action coding units proposed by Ekman and Friesen;^25^ a study by our research group showed comparable recognition performance between the VR emotions and conventional tasks of emotion recognition.

The difficulty of the exercises could be altered, for example, by making emotions more subtle, or by changing the amount of time allotted to answer. To make the software easier to use for therapists, we designed standard VR levels which gradually increased the difficulty of all parameters simultaneously (Table 1).

**Table 1.** Parameters of Virtual Reality Levels

Level	No. of stationary avatars	No. of avatars for each emotion^a^	Emotion intensity (percent)	Time emotion shown (seconds)	Time to select answer (seconds)	No. of walking avatars (distractors) in environment^b^	No. of correct answers needed to finish level (percent correct)
Practice level^c^	10	10	0	500	500	0	2 (20 Percent)
Level 1	21	3	100	60	60	3	20 (96 Percent)
Level 2	28	4	85	45	45	10	26 (93 Percent)
Level 3	28	4	75	30	30	10	26 (93 Percent)
Level 4	28	4	60	15	15	12	26 (93 Percent)
Level 5	28	4	45	10	10	12	26 (93 Percent)
Level 6	28	4	30	5	5	12	26 (93 Percent)

^a^ Six emotion profiles were used: happiness, sadness, anxiety, anger, disgust, and surprise. Finally, an emotion profile with an emotion strength of 0 percent was used, representing a neutral face. Thus, in total, seven different emotion options existed.

^b^ Due to technical limitations, the maximum number of avatars (stationary+walking) in any level was 40.

^c^ The practice level was not used in the analyses, as participants were still getting used to the VR environment in this level, and no emotions were shown (only neutral faces).

VR, virtual reality.

In the second module (sessions 6–9), participants viewed social interactions between avatars, which contained questions about the mental state of the avatar. Outside of VR, participants learned a technique to understand social situations. In the third module (sessions 10–16), participants role-played personally relevant social situations with the therapist, through VR. Outside of VR, participants learned a social problem-solving technique.

**FIG. 2.**
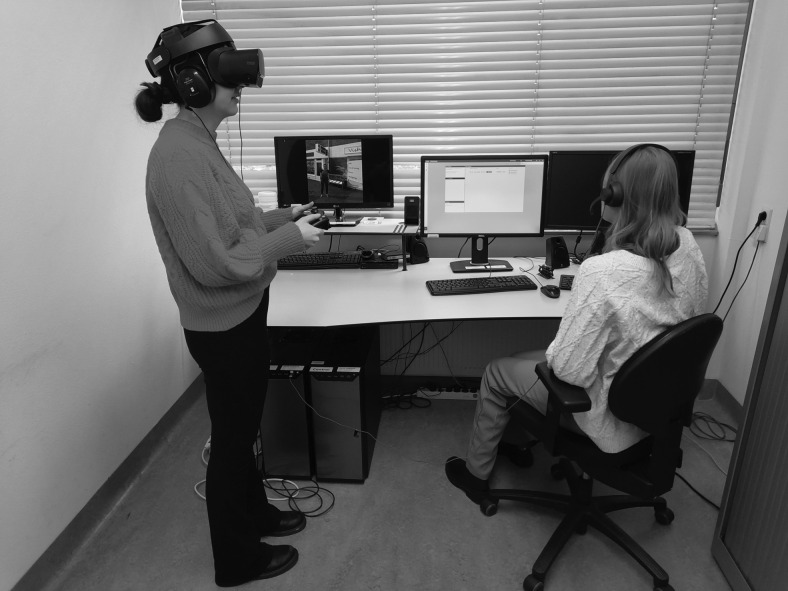
DiSCoVR hardware setup. DiSCoVR, Dynamic Interactive Social Cognition Training in Virtual Reality.

Participants explored the VR environments and selected answers using a joystick controller (Microsoft Xbox One). We used a set-up (Fig. 2) consisting of two computers and two monitors: one computer ran the VR environment, its monitor showing the participant's point of view in VR. A second, connected computer displayed the VR user interface, which was used to set up and control the VR environment by the therapist. Generally, participants used the VR headset (Oculus Rift Consumer Version 1 head-mounted display) while standing. Participants could withdraw from the VR session at any time. We did not record participants' prior exposure to VR.

### Measurements

#### Demographic, clinical, and diagnostic measures

Demographic and clinical variables were recorded in a baseline interview (see Procedure section). Premorbid IQ was estimated by administering the National Adult Reading Test^26^ (Dutch Version^27^). The number of correct pronunciations of 50 increasingly uncommon words were recorded (score range 0–100). The Mini-International Neuropsychiatric Interview Plus^22^ was administered to verify diagnoses.

#### VR measurements

The following VR parameters were available:

- Emotion expressed by the avatar.
- Answer given by the participant (correct = 1; incorrect = 0).
- VR level (Table 1).
- Response time, in seconds.
- Total time spent in the virtual environment, in seconds.

The following parameters were extracted from self-report therapist session forms:

- Treatment session dates.
- Total duration of the treatment sessions, in minutes.
- Strategies used for practice (see Supplementary Appendix SA1 for a list of standard strategies).

#### Emotion perception

The Ekman 60 Faces Test^28^ was used as a conventional emotion perception measure at baseline and post-treatment (see Procedure section). In a computerized test, participants select emotions portrayed by 60 photos (total score range 0–60).

#### Information processing

In the Trailmaking Test,^29^ participants connect the circles with numbers (TMT-A) or numbers and letters (TMT-B) in the correct order. The task completion time (in seconds) is recorded. A TMT-B score corrected for TMT-A was calculated (TMT-B divided by TMT-A).

#### Positive and negative symptoms

The positive (7 items, score range 1–7) and negative (7 items, score range 1–7) subscales of the Positive and Negative Syndrome Scale (PANSS^30^) semi-structured interview were used to assess psychotic and negative symptoms.

### Procedure

Participants expressing interest in the study were contacted by the research team. After a week of consideration, written informed consent was signed, the baseline assessment (T_0_) meeting took place, and DiSCoVR started. Within 2 weeks after the last session, participants completed the post-treatment measurement (T_1_). Both studies were approved by the Medical Ethical Committee of the University Medical Center Groningen (pilot: ABR NL55477.042.16, METc 2016/050; RCT: ABR NL63206.042.17, METc 2017/573).

### Analysis

We explored the data using descriptive statistics and visualization using the ggplot2 R package.^31^ We used only outcome data from session 2 and level 1 onward for analysis.

Each research question was analyzed with a mixed effects generalized linear model (ME-GLM), with treatment session at level 1, and participant at level 2. All models included the predictors treatment session (with session 2 coded as 0, range 0–3) and VR level (with level 1 coded as 0, range 0–5, to account for the difficulty level of the stimuli).

In the models for RQ1a and RQ1b, we also included the emotion shown (using “happy” as the reference category since it is generally recognized best^28^). In the models for RQ2a-b and RQ3, we were interested in the general change over time and predictors thereof, rather than specific accuracy of the various emotions, and therefore refrained from including the emotions shown.

The candidate models were defined using the following attributes: linear or quadratic predictors (of treatment session and VR level) and random effects structures (i.e., random slopes and/or intercepts). To estimate the ME-GLM, we used the R packages lme4^32^ (for regression model estimation) and lmerTest^33^ (for *p* values). The significance level was set at *α* = 0.05.

For RQ1a, using a logistic ME-GLM, the odds of correctly identifying an emotion were predicted by the treatment session (linear effect), the VR level practiced (quadratic effect), and the emotion shown. For RQ1b, only correct trials were modelled. Using a ME-GLM with a Gamma distribution with a log link function, the response time (in seconds) was predicted by the treatment session (linear effect), the VR level (quadratic effect), and the emotion shown.

For RQ2a-b, we fitted a logistic ME-GLM for each of the participant characteristics (RQ2a) and treatment characteristics (RQ2b) of interest. We refrained from modelling these characteristics jointly in a single model, because of multicollinearity issues. The odds of correctly identifying an emotion were predicted by the treatment session, the VR level practiced (quadratic), the participant or treatment characteristic (to estimate the main effect of the predictor), and the interaction between the participant or treatment characteristic and treatment session (to evaluate moderation of the predictor of treatment session effects). A Benjamini & Hochberg or false discovery rate correction was applied to the *p* values, to correct for multiple tests.

For RQ3, using a logistic ME-GLM, the odds of correctly identifying an emotion were predicted by the treatment session, the VR level practiced (quadratic effect), the baseline Ekman 60 Faces Test score, and an interaction between the difference score (T_1_–T_0_) on Ekman 60 Faces Test and treatment session. To investigate whether an improvement in the Ekman 60 Faces Test score was present, we conducted a paired *t* test.

## Results

### Descriptives

Demographic and clinical characteristics, as well as descriptive statistics of the VR task and treatment sessions are shown in Table 2.

**Table 2.** Descriptive Statistics (Demographic, Clinical, Virtual Reality Task Data, Treatment Session Data)

Demographic or clinical characteristic	n	Mean	SD or percent
Age	55	36.6	10.5
Gender			
Female	16		29.1 Percent
Male	39		70.9 Percent
Education level			
<Vocational	4		7.3 Percent
Vocational	30		54.5 Percent
Secondary	17		30.9 Percent
University	4		7.3 Percent
Paid employment			
Employed	12		22.2 Percent
Unemployed	42		77.8 Percent
Hours worked (weeks)	54	3.3	7.3
Work history (years)	35	6.5	6.4
Hospitalizations	49	2.7	3.6
Illness duration	53	12.4	10
Premorbid intelligence			
NLV (NART)	55	79.3	11.2
Baseline emotion perception			
Ekman 60 Faces Test	55	45.5	6.7
Baseline negative symptoms			
PANSS-N	52	16.5	5.8
Baseline positive symptoms			
PANSS-P	53	15.2	5
Baseline information processing			
TMT-A	55	37.4	13.3
TMT-B	54	90.9	40.1
TMT-B, corrected for A	41	2.4	0.9
*VR descriptives*	n *(trials)*	*Mean*	SD *or percent*
Percentage correct			
Session 2	719		78 Percent
Session 3	1,204		78 Percent
Session 4	1,535		70 Percent
Session 5	1,685		63 Percent
VR Level (1–6), mean level practiced in session			
Session 2	719	1.44	0.77
Session 3	1.204	2.61	1.01
Session 4	1,535	3.68	1.16
Session 5	1,685	4.63	1.16
Response time,^a^ overall (correct, incorrect and timed out answers)			
Session 2	719	15.08	14.74
Session 3	1,204	11.3	11.36
Session 4	1,535	9.31	9.72
Session 5	1,685	6.52	6.81
Response time,^a^ correct answers			
Session 2	558	12.97	12.15
Session 3	935	9.29	8.74
Session 4	1,073	7.94	8.17
Session 5	1,055	5.76	5.38
Response time,^a^ incorrect answers			
Session 2	137	15.9	13.29
Session 3	204	12.51	9.75
Session 4	299	8.94	8.96
Session 5	402	5.98	5.12
Response time,^a^ timed out answers (exceeded allotted response time)			
Session 2	24	59.38	3.06
Session 3	65	36.47	17.38
Session 4	163	19.02	14.03
Session 5	228	10.99	11.84
*Treatment descriptives*	n	*Mean*	SD *or percent*
Time taken to complete sessions 1–5, in days	47	19.8	8.9
Total VR Time (sessions 1–5), in minutes	55	70.3	23.5
Total session duration (sessions 1–5), in minutes	43	207.4	53.0
Number of distinct strategies used	51	2.5	1.1
Total number of strategies used (i.e., counting duplicates)	51	5.1	2.4
Percentage of sessions where strategy was used			
Verbalizing facial features	186		75.3 Percent
Mimicry	186		28.5 Percent
Attending to body language	186		15.6 Percent
Summarizing the situation	186		2.2 Percent
Attending to (the tone of) voice	186		4.3 Percent
Verifying emotion with another person	186		1.6 Percent
Thinking of a reason or context for an emotion	186		4.8 Percent
Considering how you would feel	186		2.2 Percent
Other/Custom strategy	186		1.1 Percent

^a^ In seconds.

NART, National Adult Reading Test; NLV, Nederlandse Leestest voor Volwassenen; PANSS-N, positive and negative syndrome scale, negative symptoms subscale; PANSS-P, positive and negative syndrome scale, positive symptoms subscale; *SD*, standard deviation; TMT, Trailmaking Test.

### Recognition accuracy over time (RQ1a)

**FIG. 3.**
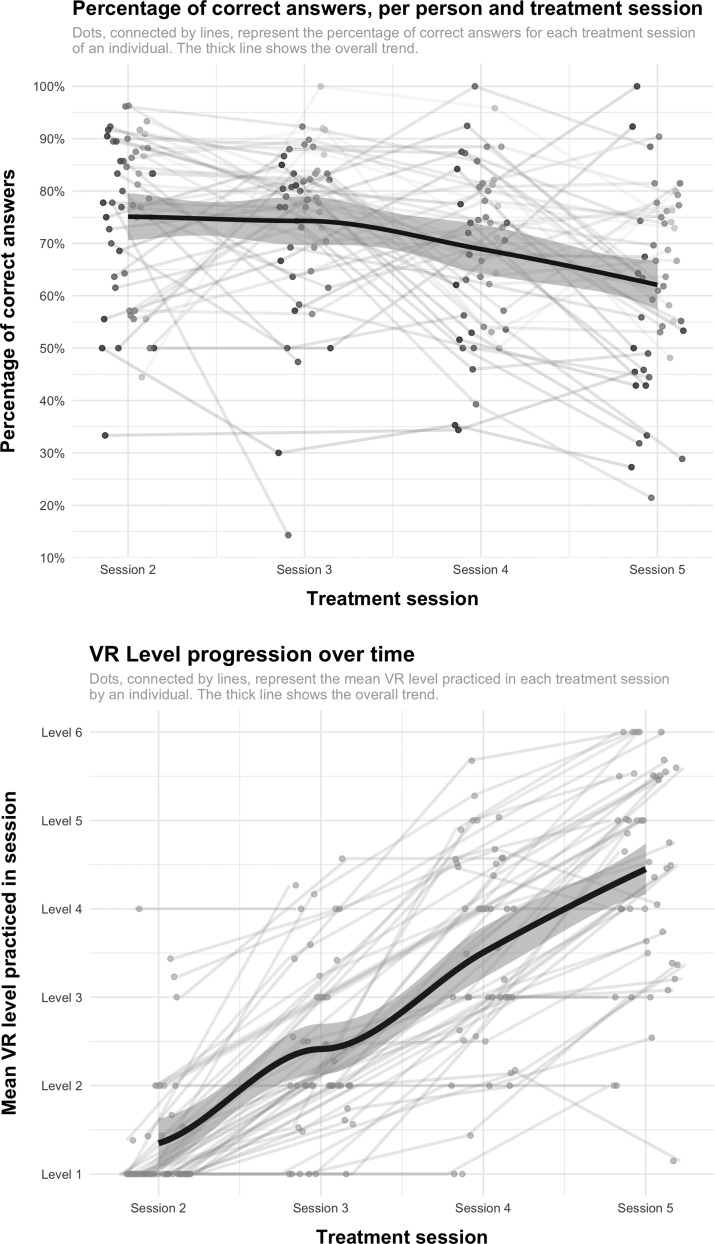
VR parameters over time (percentage of correct answers and mean level practiced).

Plots of performance over time are shown in Figure 3. The first panel shows a moderate to high percentage of correct answers (>50 percent), with variation over individuals and over time. Overall accuracy decreased over time. The second panel shows (mean) VR level progression. The plot suggests an association between treatment session and VR level; the repeated measures correlation was 0.77 (*p* < 0.001). Together, the plots suggest that participants practiced more difficult levels as sessions progressed, at the cost of a slight decrease in accuracy.

The logistic mixed-effects regression model (Table 3) showed a significant effect on response accuracy of treatment session, VR level (quadratic), and the emotions anxious, angry, sad, surprised, and disgusted. Thus, all other things being equal, the odds of correctly identifying a given emotion increased as the treatment sessions progressed and decreased as the level increased. Compared with happy faces, the odds of correctly identifying anxious, angry, sad, and disgusted faces were significantly lower, but significantly higher for surprised faces.

**Table 3.** Fixed Regression Coefficients and Test Parameters of the Mixed Effects Generalized Linear Models (for RQ1a, RQ1b, RQ2a-b, RQ3)

RQ	Model	Term	b	SE	z/t	p	
1a	Correct emotion identification	Intercept	1.77	0.15	11.75	<0.001^*^	
		Treatment session	0.20	0.06	3.54	<0.001^*^	
		VR level (squared)	−0.10	0.01	−12.22	<0.001^*^	
		Emotion: Anxious	−1.05	0.12	−8.52	<0.001^*^	
		Emotion: Angry	−0.45	0.13	−3.55	<0.001^*^	
		Emotion: Neutral	−0.11	0.13	−0.86	0.389	
		Emotion: Sad	−0.51	0.13	−3.97	<0.001^*^	
		Emotion: Surprise	0.53	0.14	3.68	<0.001^*^	
		Emotion: Disgust	−0.85	0.12	−6.86	<0.001^*^	
1b	Reaction Time of Correct trials	Intercept	2.34	0.06	39.72	<0.001^*^	
		Treatment session	−0.10	0.03	−3.67	<0.001^*^	
		VR level (squared)	−0.04	0.00	−9.72	<0.001^*^	
		Emotion: Anxious	0.35	0.04	9.52	<0.001^*^	
		Emotion: Angry	0.12	0.04	3.41	<0.001^*^	
		Emotion: Neutral	0.15	0.03	4.38	<0.001^*^	
		Emotion: Sad	0.18	0.04	5.15	<0.001^*^	
		Emotion: Surprise	0.00	0.03	0.09	0.929	
		Emotion: Disgust	0.33	0.04	9.04	<0.001^*^	
	*Covariate model* ^a,b^	*Term*	b	SE	z	p^c^	p′^d^
2a	NLV/NART	Main effect NLV	0.01	0.12	0.07	0.947	0.947
		Interaction with treatment session	−0.03	0.04	−0.87	0.387	0.781
2a	Age, squared	Main effect age	−0.34	0.09	−3.62	0.000	0.009^*^
		Interaction with treatment session	−0.03	0.03	−0.72	0.472	0.786
2a	Education level	Main effect education level	0.03	0.11	0.27	0.790	0.947
		Interaction with treatment session	0.06	0.04	1.77	0.077	0.383
2a	Gender	Main effect (Gender: Male)	−0.03	0.25	−0.10	0.917	0.947
		Interaction with treatment session	−0.07	0.08	−0.89	0.374	0.781
2a	Hospitalizations, squared	Main effect hospitalizations	−0.10	0.12	−0.77	0.443	0.781
		Interaction with treatment session	−0.01	0.04	−0.33	0.745	0.947
2a	Trailmaking A (T_0_)	Main effect TMT-A	−0.20	0.11	−1.78	0.075	0.383
		Interaction with treatment session	−0.01	0.04	−0.21	0.836	0.947
2a	Trailmaking B, Corrected for A (T_0_), squared	Main effect TMT-B (corrected for A)	0.02	0.03	0.67	0.502	0.793
		Interaction with treatment session	0.00	0.01	−0.46	0.645	0.922
2a	Ekman 60 Faces Test (T_0_)	Main effect Ekman 60 Faces	0.19	0.10	1.82	0.068	0.383
		Interaction with treatment session	0.01	0.03	0.33	0.743	0.947
2a	PANSS, Positive (T_0_)	Main effect PANSS-P	−0.11	0.12	−0.99	0.321	0.781
		Interaction with treatment session	−0.03	0.04	−0.78	0.437	0.781
2a	PANSS, Negative (T_0_)	Main effect PANSS-N	−0.20	0.12	−1.70	0.089	0.383
		Interaction with treatment session	0.00	0.04	0.13	0.896	0.947
2b	Therapy duration in days	Main effect therapy duration	0.13	0.13	1.05	0.294	0.781
		Interaction with treatment session	0.04	0.04	1.02	0.309	0.781
2b	Total session duration (1–5) in minutes	Main effect total session duration	−0.18	0.10	−1.76	0.079	0.383
		Interaction with treatment session	0.00	0.04	−0.08	0.936	0.947
2b	Total VR time in minutes	Main effect total VR time	−0.13	0.10	−1.25	0.213	0.710
		Interaction with treatment session	0.02	0.04	0.47	0.637	0.922
2b	Total strategies used, squared	Main effect total strategies used	−0.24	0.11	−2.13	0.034	0.383
		Interaction with treatment session	0.00	0.03	0.14	0.890	0.947
2b	Distinct strategies used	Main effect distinct strategies used	−0.17	0.11	−1.53	0.126	0.471
		Interaction with treatment session	−0.03	0.03	−0.82	0.411	0.781
*RQ*	*Model*	*Term*	b	SE	z/t	p	
3	Correct emotion identification, predicted by baseline (non-VR) emotion perception, and interaction of treatment session and (non-VR) changes in emotion perception	Intercept	−0.70	0.77	−0.90	0.367	
		Treatment session	0.17	0.06	2.68	0.007^*^	
		Baseline Ekman 60 Faces Test total score	0.04	0.02	2.73	0.006^*^	
		VR level (squared)	−0.10	0.01	−11.12	0.000^*^	
		Difference score Ekman 60 Faces Test	−0.01	0.03	−0.24	0.809	
		Treatment session × difference score Ekman 60 Faces Test (post-treatment − baseline)	0.01	0.01	0.76	0.446	

^a^ All covariates were scaled. If a squared covariate was used, it was squared first, then scaled, to facilitate model estimation.

^b^ Covariate models represent separate mixed-effects regression analyses; only main effects of covariates and interactions with treatment session (fixed effects) are shown in this table. The intercept and effects of treatment session (linear) and VR level (squared) were included in the models, but have been omitted from this table for the sake of parsimony.

^c^ Unadjusted *p* value of model parameter.

^d^ *p* Value of model parameter after applying an FDR correction.

^*^ Significant at α = 0.05.

FDR, false discovery rate; PANSS, Positive and Negative Syndrome Scale; *SE*, standard error.

### Emotion recognition reaction speed over time (RQ1b)

**FIG. 4.**
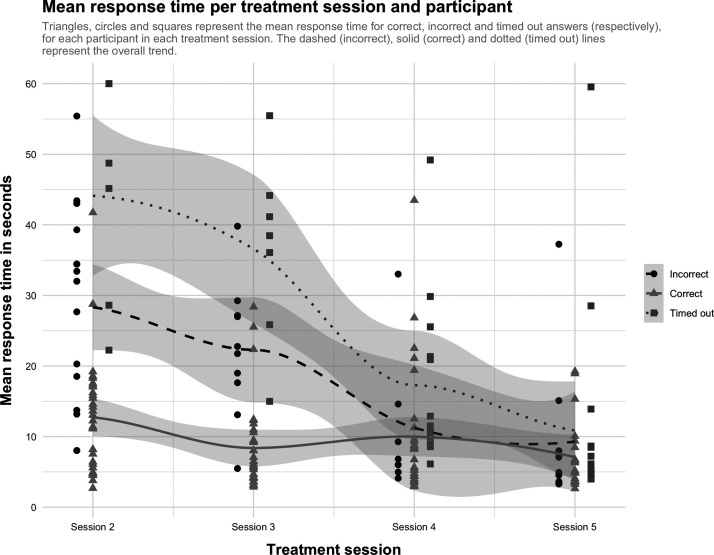
Response time across treatment sessions (for correct, incorrect, and timed out answers).

A plot of mean response time for correct, incorrect, and timed out responses over time is shown in Figure 4. As is shown in Table 2, response times decreased over time. The results of the generalized linear model using a Gamma distribution (Table 3) showed that, all other things being equal, response times for correct answers *decreased* as treatment sessions progressed and *increased* with level progression. Response times were longer than for happiness for all emotions, except surprise.

### Participant and treatment predictors of recognition accuracy (RQ2a-b)

The results of the regression analyses investigating moderators of treatment session effects are shown in Table 3. After correction, there was only a significant main effect of age (quadratic and scaled): as age increased, the overall odds of correct identification decreased (cf. Supplementary Fig. S1 in Supplementary Appendix SA3). No interactions between any of the investigated variables and treatment session were found.

### Performance on VR task versus conventional task (Ekman 60 Faces) (RQ3)

A paired *t* test showed a significant improvement in the total Ekman 60 Faces Test score (*t* = 2.72, df = 43, *p* = 0.009) from baseline to post-treatment. The model for RQ3 in Table 3 shows that the T_0_ Ekman 60 Faces Test score was a significant predictor of correct emotion identification. However, there was no significant interaction between treatment session and Ekman 60 Faces Test difference score, suggesting that improvement across treatment sessions was not different for people with higher or lower Ekman 60 Faces Test difference scores.

## Discussion

### Main findings

We found that while accounting for the difficulty level of the exercises and the specific emotion to be recognized, emotion perception performance on a VR task improved as treatment progressed. Participants also provided faster correct responses over time. We found no evidence of moderation of treatment or participant characteristics of treatment effects.

Finally, baseline emotion recognition performance on a photo task (Ekman 60 Faces) predicted correct emotion identification in VR, but there was no evidence that people who benefited more from VR training, also showed larger improvement on the Ekman 60 Faces task.

### Emotion recognition in VR

We did not find that improvement in VR was associated with improvement outside VR, suggesting a discrepancy between the VR emotion recognition task and non-VR tasks of social cognition. The question, therefore, remains to what degree improvements in VR generalize to performance on other social cognitive tasks and daily life. As pointed out in the Introduction, a study^18^ using the same VR environment found that performance and confusion patterns across the VR task, a photo task (Ekman 60 Faces Test), and a video task^34^ were comparable in healthy individuals.

This is in line with our finding that baseline Ekman 60 Faces scores were predictive of VR emotion recognition accuracy. While this suggests that the VR task tapped into similar skills as conventional social cognition tasks, relationships between performance on the different tasks were not directly investigated in the aforementioned study.

The lack of an interaction between improvement in VR and change in emotion perception outside VR suggests that DiSCoVR may have had limited generalizability, a possible reason for the lack of effects of DiSCoVR in our RCT.^11^ It is possible that, due to graphical limitations, the way emotions are currently rendered in VR is too simplistic, and therefore not applicable to real-life emotions. For example, it has repeatedly been found that the emotion disgust is recognized better on photographs of faces than in VR.^19,35^

Ultimately, VR emotions are a simplification, and may not (yet) adequately represent the complexity of real emotions.^19,36^ Graphical limitations aside, it is also possible that the VR exercises did not adequately simulate real-life social interactions, in which people are a participant rather than an observer and a personally relevant social context is present. These components of social interactions have been proposed as essential qualities of ecologically valid social cognition tasks;^37^ without them, the added value of using VR to practice may be limited.

Given that the widespread adoption of VR as a therapeutic tool is relatively recent, only a few studies targeting social interaction difficulties in psychotic disorders are available investigating the added value of VR by comparing VR treatment with non-VR treatment. Tsang and Man^38^ (*n* = 95) compared VR vocational rehabilitation training with a non-VR, therapist-led equivalent and conventional vocational training and found that VR led to greater improvement in cognition, but the analog format led to greater improvement in a face-to-face work performance test.

Park et al.^39^ compared VR social skills training to conventional social skills training, and found that VR training led to greater motivation, improvements in conversational skills and assertiveness, but conventional training had a greater effect on expressive nonverbal skills.

No clear patterns have, therefore, emerged regarding target processes that are more effectively improved by interventions using VR than by a non-VR equivalent. A blended approach is possibly necessary, where emotion recognition practice occurs both in VR and in real-life social situations.

### Moderators of improvement in VR

We found a main effect of age on emotion recognition accuracy, replicating other studies.^18,19^ Contrary to previous meta-analyses on (non-VR) SCT finding associations between emotion perception and gender, hospitalization status, clinical symptoms and antipsychotic treatment,^20^ we found no other predictors or moderators of accuracy. This could be due to the absence of an effect, interference due to the interrelatedness of time and task difficulty, and/or insufficient power to detect moderation effects.

On the other hand, our results regarding moderators of treatment effects are consistent with previous meta-analyses.^6,7,40^ Thus, while more research is needed regarding the optimal approach to (VR) emotion recognition training, the present evidence suggests it can be beneficial regardless of the exact training parameters (e.g., session duration) and participants' demographic and clinical characteristics.

### Strengths and limitations

This study is the first to investigate the learning process of participants engaging in emotion recognition training, and SCT in general. It is also the first to use VR for this purpose. Given that the primary purpose of the sessions investigated presently was therapeutical, these data provide a relatively accurate picture of how SCT might work in a clinical setting.

However, the process of data collection was not optimal for research, as data collection was not the main goal of the treatment sessions. Particularly, the interrelatedness of treatment session and VR level makes it difficult to disentangle the effects of difficulty versus time. Further, data from two separate studies were combined. We cannot exclude the possibility that impactful differences existed between the studies.

### Implications and suggestions for future research

Our results suggest that external validity could be an issue for DiSCoVR, and possibly VR emotion recognition training in general. Given that ecological validity is regarded as one of the main benefits of VR, it is vital to investigate further how improvements in performance in VR relate to performance on other social cognitive tasks and real-world social functioning.

Our results may imply that VR emotions do not sufficiently resemble real facial emotions. Technological advancements in facial rendering and VR resolution may partially address this issue. Future research could study improvement in facial expression through adding micro-expressions, mixed emotions and individual variability in emotional expression.

In addition, complementing VR-SCT with additional types of practice material (e.g., photos, videos) could help participants develop a broader range of recognition skills. Moreover, studies should investigate the strengths and weaknesses of VR-SCT compared with conventional training, to identify for which target processes VR can be used successfully.

It is possible that VR is not (yet) appropriate for targeting complex perceptual processes requiring highly realistic VR stimuli. For other treatment targets, however, VR interventions have repeatedly been shown to be effective and generalizable. For example, RCTs on VR cognitive behavioral therapy and exposure have found improvement in social participation, persecutory delusions, and hallucinations.^17,41–43^ Thus, targeting emotional or cognitive experiences with VR could (currently) lead to greater generalization.

Finally, this study demonstrated the value of investigating treatment processes *during* treatment. Future studies could investigate additional parameters during interventions, such as gaze and approach versus avoidance behavior. This could be supplemented with experience sampling methods to investigate relevant processes in daily life. This way, procedural changes during treatment can be investigated, and VR tasks and treatments potentially further improved.

## Supplementary Material

Supplemental data

Supplemental data

Supplemental data

## Supplementary Material

Supplementary Appendix SA1

Supplementary Appendix SA2

Supplementary Appendix SA3
