# Supplementary material for: Trajectories of Emotion Recognition Training in Virtual Reality and Predictors of Improvement for People with a Psychotic Disorder
Source: Cyberpsychol Behav Soc Netw. 2023 Apr 14;26(4):288–99. doi: 10.1089/cyber.2022.0228 (PMC10125400; doi:10.1089/cyber.2022.0228)
Supplement: Supplemental data [file Supp_AppS1.docx]

# Appendix 1: Strategies

***The strategies were presented to participants on the following worksheet:***

To identify how someone else is feeling, or what he or she thinks or wants, you can use different tricks or methods (strategies). A few examples are:

| **Features**  Pay attention to facial features (eyes, mouth, eyebrows, cheeks, etc.) | **Mimic emotions**  Mimic emotions, so you can experience what an emotion feels like with your own face. | **Body language**  Look at somebody’s posture/gestures/body language. | **Voice**  Pay attention to what the other person’s voice sounds like (pitch, speed, volume). |
| --- | --- | --- | --- |
| **Compare situation**  Compare the situation to something you've experienced, and consider how you felt in this situation. | **How would you feel?**  Consider how you would feel in the same situation. | **Verify emotion**  Ask somebody how they’re feeling, for example, by saying “How do you feel about this?" | **Ask someone else**  Ask a third party / outsider (e.g., a friend) how they think someone else is feeling. |
| **Compare to previous experiences**  Compare to previous experiences you've had with this person or similar situations. | **How would another person feel?**  Ask someone how they would feel in a certain situation. | **Summarize**  Briefly summarize the situation for yourself and list the most important facts. | **Am I correct in my beliefs?**  Ask yourself about every ‘fact' whether this is actually a fact, or how you have interpreted the situation. What did you base your interpretation on? Is that correct? |

Of course, these are just a few of the possible strategies to use in a social situation. Which strategies do you personally use?

Strategies are not always useful in every single situation. For example, if you are on the phone, it is difficult to mimic the other person’s emotions (since you can’t see them). And if you haven’t experienced a situation, you cannot always compare it to something you've personally experienced. That is why it is a good thing to practice multiple strategies.

In conversations, you could use one or more of the following strategies:

| **Summarize & Check**  Summarize what the other person has said, and ask whether you’ve understood them correctly. | **Ask the other person**  In a situation where you’re in doubt what the intentions of the other person are, ask them what they think is the best way to approach something. | **How would you react?**  Ask another person how they would react in a certain situation. |
| --- | --- | --- |
| **Time-out**  Give yourself a ‘time out’ (for example: briefly look at your phone, or go to the bathroom) so you have some time to gather your thoughts. | **Cue**  With friends, your partner or a family member, think of a (secret) cue to use in social situations when you’re not sure what’s going on, so that they can help you (in a subtle way). | **Check**  Before you do or say something, ask yourself what is going on in the other person’s mind, and why they are behaving in a certain way. |
| **Is it helpful?**  Before you do or say something, ask yourself: Does this behaviour help, or does it only make things worse? | **Make a list**  Make a list of possible ways to react, and choose the best option. | **Role-play**  Practice situations you find difficult in a role-play, for example with a friend, a family member or a clinician. |
