# Supplementary material for: Trajectories of Emotion Recognition Training in Virtual Reality and Predictors of Improvement for People with a Psychotic Disorder
Source: Cyberpsychol Behav Soc Netw. 2023 Apr 14;26(4):288–99. doi: 10.1089/cyber.2022.0228 (PMC10125400; doi:10.1089/cyber.2022.0228)
Supplement: Supplemental data [file Supp_AppS2.docx]

# Appendix 2: Homework Assignment Module 1

## Emotions in my daily life

Think of a situation that you’ve experienced today with somebody else. Write down which emotions the other person expressed, and how you identified them.

| **Emotion**  **(Happy, angry, sad, anxious, surprised, disgusted)** | **Situation** | **How did you identify the emotion?**  **Which strategy did you use?** |
| --- | --- | --- |
|  |  |  |
|  |  |  |
|  |  |  |
|  |  |  |
|  |  |  |
