# Supplementary material for: Trajectories of Emotion Recognition Training in Virtual Reality and Predictors of Improvement for People with a Psychotic Disorder
Source: Cyberpsychol Behav Soc Netw. 2023 Apr 14;26(4):288–99. doi: 10.1089/cyber.2022.0228 (PMC10125400; doi:10.1089/cyber.2022.0228)

# Appendix 3: Percentage of correct answers by age

Supplemental Figure 1: Percentage of correct answers (overall), by age.


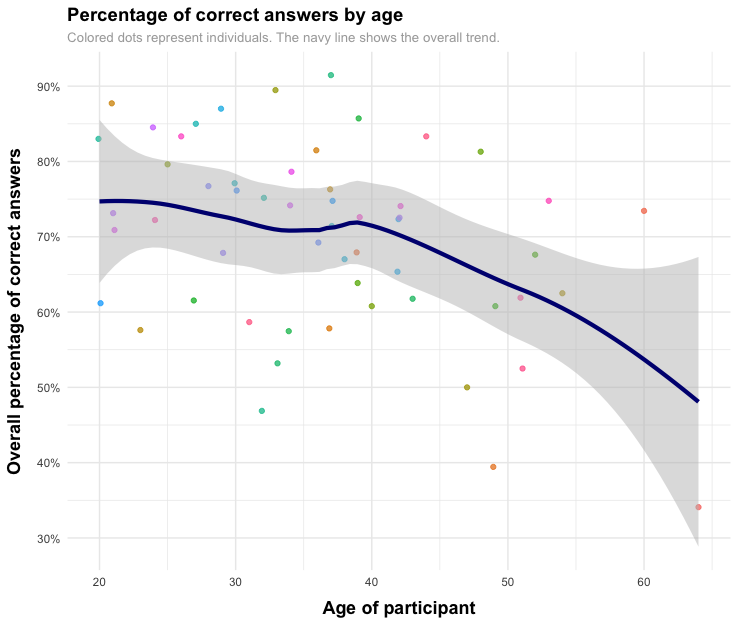

Supplement: Supplemental data [file Supp_AppS3.docx]
